# Impact of Repeated Exposures on Information Spreading in Social Networks

**DOI:** 10.1371/journal.pone.0140556

**Published:** 2015-10-14

**Authors:** Cangqi Zhou, Qianchuan Zhao, Wenbo Lu

**Affiliations:** Center for Intelligent and Networked Systems (CFINS), Department of Automation and TNList, Tsinghua University, Beijing, 100084, China; Beijing University of Posts and Telecommunications, CHINA

## Abstract

Clustered structure of social networks provides the chances of repeated exposures to carriers with similar information. It is commonly believed that the impact of repeated exposures on the spreading of information is nontrivial. Does this effect increase the probability that an individual forwards a message in social networks? If so, to what extent does this effect influence people’s decisions on whether or not to spread information? Based on a large-scale microblogging data set, which logs the message spreading processes and users’ forwarding activities, we conduct a data-driven analysis to explore the answer to the above questions. The results show that an overwhelming majority of message samples are more probable to be forwarded under repeated exposures, compared to those under only a single exposure. For those message samples that cover various topics, we observe a relatively fixed, topic-independent multiplier of the willingness of spreading when repeated exposures occur, regardless of the differences in network structure. We believe that this finding reflects average people’s intrinsic psychological gain under repeated stimuli. Hence, it makes sense that the gain is associated with personal response behavior, rather than network structure. Moreover, we find that the gain is robust against the change of message popularity. This finding supports that there exists a relatively fixed gain brought by repeated exposures. Based on the above findings, we propose a parsimonious model to predict the saturated numbers of forwarding activities of messages. Our work could contribute to better understandings of behavioral psychology and social media analytics.

## Introduction

With advances in information and communication technology, more and more people around the world are getting involved in online social networks. In using these services, a simple click will forward a message to another group of people and this simple action significantly facilitates the spreading of information [1, 2]. Information spreading in social networks has long been an active research area. What factors make an individual spread a message by clicking the forward button is still an open question [3–5]. Apart from the intrinsic interest orientation [6], the daily routine schedule and the information processing ability of oneself [7, 8], could other people’s behavior affect ones decisions? In real life, there exists the phenomenon that information, even if it is unfounded, will probably be accepted if enough individuals repeatedly mentioned it. Also, people tend to change their thoughts or habits by taking advice from their friends [9]. These phenomena reflect the interactions among people when they are making decisions. A direct and simple consequence of these interactions appears to be the increase in the chances of repeated exposures to similar information, due to triadic closure patterns in social networks [10]. A better understanding of the effect of repeated exposures could bring more social and economic benefits. For instance, repeated exposures could bring positive impact on public health by changing TV viewers’ knowledge, attitudes and behaviors [11]; repeated person-to-person recommendations will increase the probability of buying [12]; the impact of repeated exposures on information spreading in microblogging services is nontrivial [13, 14]. More examples could be found in [15]. These works demonstrate the significance of repeated exposures to information carriers for spreading. The impact of this effect is, however, not clearly elucidated in a lot of research fields. In this paper, we aim to find out if repeated chances of exposures will actually increase people’s willingness to spread information and furthermore, to what extent this effect plays the role for spreading information. We believe that the answers to these questions will shed light on how people traverse their psychological thresholds to approve new information or adopt new innovations, and on how to utilize the topology of networks for spreading enhancement and viral marketing [16].

We aim to conduct a topic-independent research. We hypothesize that people’s attitudes toward the contents of information reflect the quality of their intrinsic interest, and that these attitudes are relatively stable with respect to specialized contents within a short period of time. Hence, the topology of networks only provides a precondition for this effect to happen. Based on the above hypotheses, people’s responses toward the messages which cover sufficiently various topics should show similar characteristics in the networks with statistically different and diverse enough microscopic structure. This is because sufficiently various contents could reflect average interest of a group of people. Moreover, people’s attitudes toward different types of topics should be different [13]. For instance, the impact brought by commercial advertisements should be differently evaluated compared with the impact brought by insignificant grumbles from celebrities.

In order to validate the above hypotheses, based on the data collected from a popular microblogging service, we conduct a large-scale analysis to explore the answer to our questions. In microblogging services, people are connected with each other by *following* and these connections form huge and complex networks. These online networks, with detailed records of people’s activities, give access for researchers to explore traditional behavioral and psychological problems with unprecedented data sets [17–20]. After a message is posted, it can be exposed to users either once or multiple times. It is difficult to precisely identify how many times an individual actually sees a message. However, when a message has been repeatedly pushed to a user, we can ensure that the user has a relatively high probability to see this message multiple times. Hence, we mitigate this problem by considering forwarding activities under repeated exposures rather than under certain numbers of exposures. Furthermore, we propose the definition of *direct follower networks* to reduce the scale of our problems. The scope of the spreading of our message samples can be iteratively decomposed into several direct follower networks, and we observe that only the networks of influential root users that remarkably contribute to the spreading of information. Specifically, we analyze the ratios of the forwarding probabilities under repeated exposures to that under only one exposure. We also demonstrate the robustness of the ratios against the change of message popularity. Our findings are simple, and they provide us a rule of thumb to predict the saturated numbers messages have been forwarded. Our prediction is relatively accurate for those messages with large numbers of forwarding activities.

## Related Work

### Social Reinforcement

The effect of social reinforcement is relevant to our research [21]. Social reinforcement means that people need repeated confirmations from their neighbors to adopt new things or take their own actions. The so-called complex contagion requires multiple sources to trigger adoption, while its counterpart, simple contagion requires only one contact to trigger adoption regardless of the number of times required for a success in a Bernoulli-like sequence of trials. Due to this difference, traditional epidemic models are not suitable for complex contagion [22]. Centola [23] conducted a controlled experiment to show how clustered network structure affects complex contagion. It turns out that social behaviors spread more widely and quickly in clustered networks than in random networks. Several studies extend the research of this problem in specific areas. Gonzalez-Bailon et al. [24] studied the dynamics of protest recruitment in Twitter. Their work also supports the concept of complex contagion with evidence from real data. Ver Steeg et al. [25] performed an empirical study to discover what factors affect the speed of the spreading of social contagion. They found that clustered structure combined with the marginal effect of repeated exposures limit the size of cascades in the social media site Digg. In addition, some researchers focused on the impact of local network structure on different types of contagions. Ugander et al. [26] studied how an individual’s neighborhood structure affects social contagion. They found that the number of connected components rather than the size of the neighborhood controls the probability of contagion. Weng et al. [27] studied how complex and simple contagions permeate community structure in networks. They found that most memes spread like complex contagions, while a few are viral like diseases.

The works mentioned above center on the definition of complex contagion. Complex contagion requires an individual to expose to multiple sources, rather than to expose multiple times. This definition is subtle and is well explained in [21]. When we consider forwarding activities in microblogging, one-time exposure has no ambiguity since people must have seen the message at least once before they forward it. While repeated exposures may be the case of either actual complex contagion (such as the messages containing advertisements or obscure information which people require multiple stimuli to accept) or a sequence of Bernoulli trials (such as the messages containing news or witticisms which some people will eventually see and accept after successively missing several identical ones). Compared with previous works, we aim to come up with conclusions from large-scale data sets for elucidating the mechanism of the enhancement of spreading brought by repeated exposures, rather than limit the study on rigid definition of complex contagion. Several previous related works focused on the impact of topics or network structure on information spreading, while we intend to discover valuable invariant factors from people’s forwarding dynamics. These constant factors may reflect the nature of people’s online behaviors and could be the rule of thumb for real-world applications.

### Simulation Models

There are also some researchers who have put a lot of effort into establishing or revising simulation models to describe the role that repeated exposures play in spreading dynamics. Lü et al. [28] took into account the memory effect, the social reinforcement and the non-redundancy of contacts in their spreading model. They investigated the differences between information and epidemic spreading and the impact brought by network topologies. Krapivsky et al. [22] and Zheng et al. [29] both considered the effect of social reinforcement in establishing their simulation models. The former work accounted for this problem by endowing each individual with multiple states rather than only two before final adopting. The latter work proposed a recursion with a constant reinforcement intensity to express the approving probabilities under different numbers of exposures. These works have great theoretical value for understanding the effect of reinforcement. We intend to, however, perform an inductive study rather than a deductive one to explore how repeated exposures affect information spreading using empirical data. Our work may help to validate the accuracy and effectiveness of existing simulation models.

### Prediction Methods

Our findings allow us to predict the number of times a message will be forwarded under some conditions. The prediction task of which messages are popular or important is of great significance. We can take advantage of the popularity of messages for emergent topic detection, recommendation and viral marketing. Several previous studies formulated this task into classification problems. Hong et al. [30] investigated the features based on the contents of messages, temporal information, metadata of messages and users, as well as network structural properties. The messages with thousands of forwarding activities are well predicted by their method. Tsur et al. [31] presented an efficient method based on a linear regression model to predict the propagation of Twitter hashtags. They analyzed various feature types and concluded that content features can be used as strong predictors. Ma et al. [32] adopted five standard classification models for the prediction of the popularity of new hashtags in Twitter. They extracted content and contextual features and discovered that these features outperform the baseline methods and that the logistic regression model performs the best. Instead of formulating the prediction of the popularity of messages into a classification problem, we adopt the collective information of the dynamics of a certain message at an early stage to predict its future trend in a late stage. The reason this task can be achieved is that our findings show a simple relation between the willingness of forwarding messages under one exposure and the willingness under repeated exposures.

## Results

### Direct Follower Networks

Goel et al. [33] found that the vast majority of information spreading processes end up within one degree of the initial root user. And the patterns are similar across seven online domains. This one-degree network structure allows us to reasonably reduce the scope of the spreading of information considered in our research. And this assumes that a large amount of activities occur within one-degree of the root user. Analogous to the definition of ego network [34], we define this “wheel-like” directed network as *direct follower network*. A direct follower network is composed of the set of all one-hop followers of a certain user (the root user or the author), and the set of all directed links among them. We calculate the portions of the amount of activities in direct follower networks to the amount in the corresponding whole networks for all of our 3,506 original messages. The portions exceed 80% for 89.45% of all the samples, which means that the overwhelming majority of activities are contributed by one-hop followers of root users. Hence, the simplification is rational and we are allowed to adopt our definition for subsequent tasks.

By examining the variation patterns of the intensity of forwarding activities with respect to time, we discover that these patterns can be roughly classified into single-peak and multi-peak ones. These patterns reflect the responses of followers to their influential root users’ messages, and they could be viewed as relaxation processes to sharp *δ*-like stimuli. This phenomenon is consistent with the exogenous shocks in [35]. By utilizing the characteristics of these patterns, we are allowed to roughly distinguish single-peak patterns from multi-peak ones (see Materials and Methods). We discover that 3,349 among 3,506 samples are single-peak. The rest 157 multi-peak samples include 150 ones with 2 peaks, 6 with 3 peaks and only 1 with 4 peaks. In fact, we observe that it is basically the influential root users with large-size direct follower networks who have the abilities to stimulate sharp peaks. Hence, a single-peak pattern implies that most activities are happened in a dominant direct follower network. The fact that multi-peak patterns own no more than 4 peaks implies that the activities just diffuse to the direct follower networks of other influential users, like a relay race. So we can decompose the spreading dynamics into two or more direct follower networks for those samples with multi-peak patterns. And this definition could be a building block for related research. Base on the above analysis, it is reasonable to focus our research of information spreading on direct follower networks.

### Conditional Forwarding Probabilities

Since that direct follower networks significantly reduce the size of networks, it is convenient for us to trace dynamical processes and calculate forwarding probabilities under the conditions of either only one exposure or repeated exposures.

**Fig 1:**
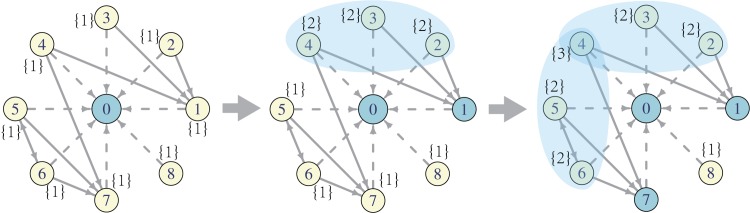
Forwarding dynamics in a direct follower network. The figure in the left shows a simple direct follower network of root user 0. When user 0 posts a message, all of his followers will be exposed to this message once (the numbers in braces). In the second step, user 1 forwards this message (dark colour), all the followers of user 1, which is user 2, 3 and 4 (in the shaded area) are exposed to this message one more time. Then user 7 forwards this message. Same thing happens except that now user 4 has been exposed to the message three times since he follows both user 1 and user 7.

Suppose there is a direct follower network *D* corresponding to a root user *u*_*r*_. Let us define *σ*_*i*_(*t*) as the number user *i* has been exposed to a message at time *t*, where the index *i* runs over all the nodes in network *D*. At the beginning, *u*_*r*_ posts a message, and all the followers of *u*_*r*_ are simultaneously exposed to the message once. Then a follower *u*_1_ forwards this message without any interaction with other followers. This random event triggers that all the followers of *u*_1_ have the chance to see this message one more time. As the number of users who forwards this message grows, all *σ*_*i*_ change monotonically and non-decreasingly. Fig 1 demonstrates the dynamics mentioned above. The decisions of forwarding the message or not made by users who are exposed to the message more than once are probably affected by other users. Hence, the value of *σ*_*i*_(*t*) of user *i* depends on whether or not the *followees* of user *i* forward this message, before time *t*.

**Fig 2:**
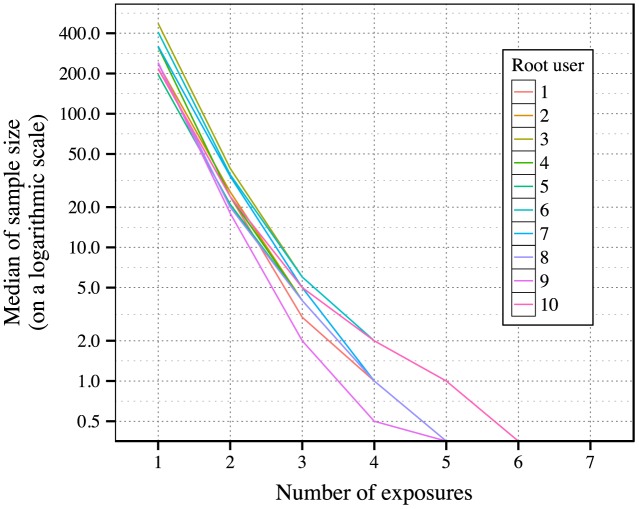
Number of users who forward messages under certain number of exposures. For the top 10 most active root users, we count the number of their followers who have forwarded their messages under certain number of exposures, respectively. In this figure, we show the median of these counts with respect to exposure number. In order to clearly show the results, the *y*-axis is transformed into a logarithmic scale. There are no remarkable forwarding activities when exposure number exceeds 5.

We aim to calculate the probabilities of users’ forwarding behavior under different numbers of exposures. We focus on two conditions: 1) one single exposure; 2) repeated exposures. The main reason we aggregate several multiple exposures into a single effect is because moderately large number of exposures happen rarely. In our data, the users who forward messages under 1, 2 and 3 exposures account for 98% of all users who forward messages. Also, the users who eventually do not forward messages under 1, 2 and 3 exposures account for 99.7% of all users who do not forward messages. We plot the sample sizes of users who forward messages under certain numbers of exposures in Fig 2 for every root user. As exposure number increases, the sample size decreases sharply. It is not feasible to obtain a forwarding probability under a certain number of exposures when the sample size is too small under this condition.

In addition, previous studies [12–14] show that the chances of adopting new information or innovations will not increase sustainably after a certain number (commonly a relatively small one) of stimuli. Later on we will also demonstrate similar results. Also, it is more likely to ensure that users have high probabilities to see a message multiple times than to identify the number users actually see a message. Hence, we only focus on two conditions (mentioned above). Instead of just comparing which chance is larger than the other under those two conditions, we also aim to discover to what extent these chances are different.

We define *S*(*t*) as the number of users who are exposed to a message only once, but do not forward it before time *t*; and *M*(*t*) as the number of users who are repeatedly exposed to a message, but do not forward it either before time *t*. We denote by *NR*(*t*) as the set of users who have not forwarded a message at time *t*. *S*(*t*) and *M*(*t*) can be represented with respect to *σ*_*i*_(*t*) as follows:

$$\begin{array}{c} S\left(t\right)=\sum_{i}I_{\sigma_{i}\left(t\right)=1},\;i\in NR\left(t\right) \end{array}$$ (1)

$$\begin{array}{c} M\left(t\right)=\sum_{i}I_{\sigma_{i}\left(t\right)>1},\;i\in NR\left(t\right) \end{array}$$ (2)

Where *I* is the indicator function. We also define *R*_*s*_(*t*) as the number of users who are exposed to a message only once and then forward it before the next exposure, and *R*_*m*_(*t*) as the number of users who are repeatedly exposed to a message and then forward it. At a certain time *t*, we define *p*_*s*_ = *R*_*s*_(*t*)/(*S*(*t*)+*R*_*s*_(*t*)) and *p*_*m*_ = *R*_*m*_(*t*)/(*M*(*t*)+*R*_*m*_(*t*)) as the probabilities of forwarding a message under the conditions of one-time exposure and repeated exposures, respectively.

**Fig 3:**
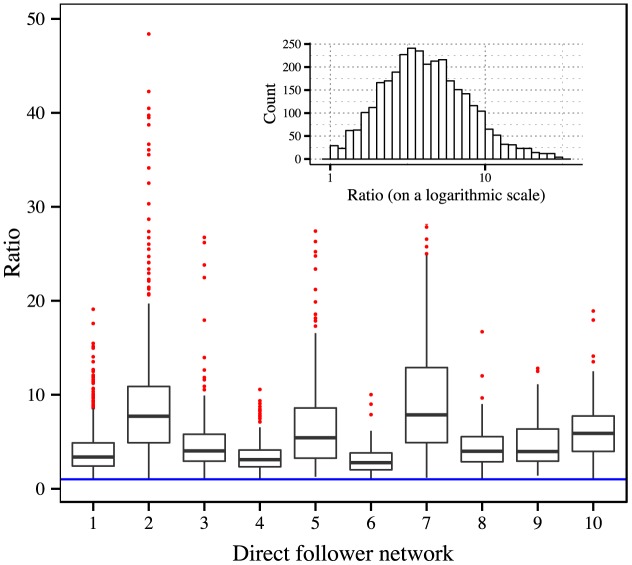
Boxplots of the distributions of *p*_*m*_/*p*_*s*_ for the top 10 most active direct follower networks. The details about these networks can be found in Materials and Methods. The blue line indicates the position of 1. The inset figure shows the histogram of all the ratios on a logarithmic scale.

We build the direct follower networks for the top 10 most active influential users, and collect the messages originally posted by them as samples to calculate *p*_*s*_ and *p*_*m*_. We find that an overwhelming majority of *p*_*m*_ are greater than *p*_*s*_ (3,225 out of 3,506 samples). For these 3,225 samples, we show the boxplots of *p*_*m*_/*p*_*s*_ for each root user in Fig 3. The distribution of *p*_*m*_/*p*_*s*_ is relatively concentrated in a small interval of [1, 7] (account for 80% of all the ratios). After carefully examining the distributions illustrated by these boxplots, we discover that except for the medians of ratios in network 2 and network 7 (these two medians exceed 7), the other 8 networks show a relatively fixed median, which is 4.070 ± 1.086 (standard deviation). And all medians of these 10 distributions are of the same order of magnitude. Interestingly, the root user of network 2 is an account for posting obscure jokes; the root user of network 7 is an account for posting messages about astrology. The messages posted by these two accounts are specialized in specific topics. By comparison, the messages posted by the other 8 root users are more diverse and mixed in contents, regardless of the differences in network structure. In addition, compared to the accounts of firms and organizations, all 5 celebrities are among the latter 8 cases. It makes sense due to the randomness and diversity of human activities.

**Fig 4:**
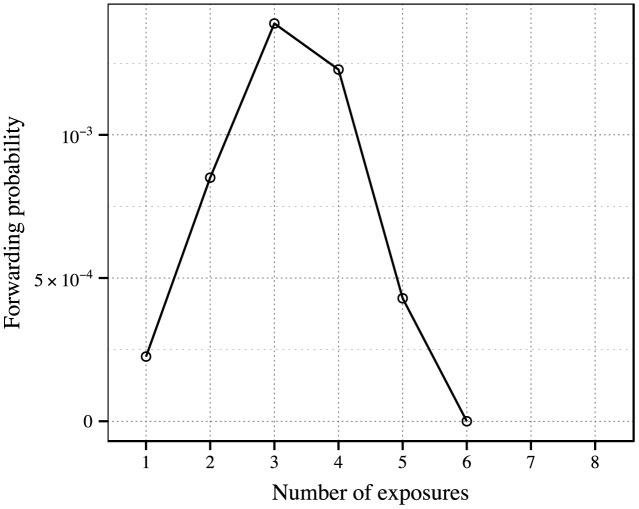
Forwarding probability under 1 to 8 exposures. Each value is the median of all the forwarding probabilities under the corresponding number of exposures. It is due to the rareness of data that the values under 7 and 8 exposures are zero.

Then we show the forwarding probabilities under certain numbers of exposures in Fig 4. Each value is obtained by taking the median of all results under a certain number of exposures. The probabilities under 2, 3 and 4 exposures are significantly higher than the probability under a single exposure. The variation pattern of the ratio of *p*_2_/*p*_*s*_, *p*_3_/*p*_*s*_, … is the same as the pattern shown in Fig 4, since the only difference between them is a multiplier of normalization constant. The sample size in our data decreases sharply as exposure number increases, and we believe that small sample size makes the separated results unreliable. We make a trade-off between detailed demonstration and the reliability of our results. And we focus on the differences between the effects of single exposure and repeated exposures.

### The Effect of Popularity

Although the medians of *p*_*m*_/*p*_*s*_ are relatively fixed in different networks, are they robust against the change of message popularity?

**Fig 5:**
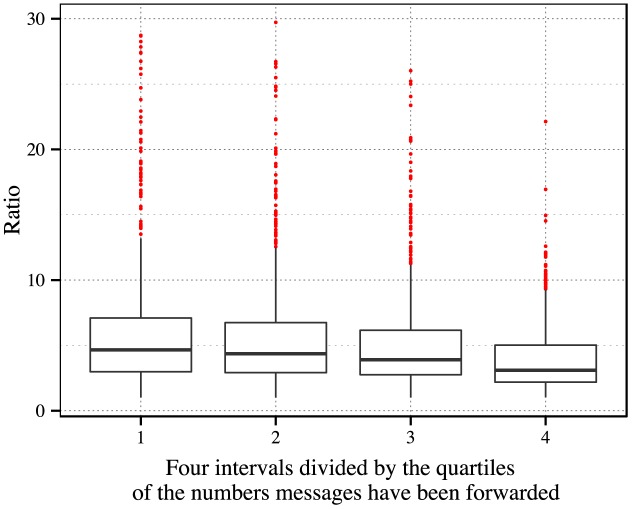
Variance of the medians of *p*_*m*_/*p*_*s*_ with respect to the level of popularity of messages. All the ratios of *p*_*m*_/*p*_*s*_ are mixed together and divided into four intervals according to the level of popularity of messages. Within these 3,225 samples, we omit 21 very large outliers for clear visualization. The level of popularity increases from the 1st interval to the 4th (see Materials and Methods for details). Boxplots are plotted for the ratios in each interval. The medians of the ratios, which are 4.654, 4.362, 3.898 and 3.100 respectively, decrease slightly as the level of popularity increases.

We use the total number a message has been forwarded as the index to represent its popularity [30]. And all the ratios of *p*_*m*_/*p*_*s*_ in all 10 networks are split into four parts by the quartiles of the numbers messages have been forwarded. Let *A*_*i*_, *i* = 1, 2, 3, 4 denotes the *i*th part of data. As shown by Fig 5, boxplots are plotted for each part. It seems that, as the level of popularity of messages changes, the medians of the distributions of *p*_*m*_/*p*_*s*_ change slightly.

In order to examine if there exist significant differences among the 4 medians demonstrated in Fig 5, we perform statistical tests of significance using two methods, permutation test and *Z*-test after logarithm transformation. The commonly used *Z*-test and *T*-test require that the data follows a normal distribution. Such strict condition limits the use of significance tests in real-world applications. Instead, we adopt a nonparametric approach which makes no normal assumption, and a transformation approach which approximately makes the data normal.

Firstly, we use one-sided permutation test as the nonparametric approach, which rearranges the labels of data points and calculates the test statistic. Let *ξ*_*i*_, *i* = 1, 2, 3, 4 be the medians of the distributions of 4 different parts of data *A*_*i*_. The *null hypothesis* *H*_0_ states that: there are no significant differences between any two *ξ*_*i*_, i.e., *ξ*_*i*_ = *ξ*_*j*_, *i* ≠ *j*. We combine every pair of data sets, rearrange the labels of data points and calculate *p*-value, which is the portion of simulations yielding more extreme statistics than the observed one in our data. The *p*-values are shown in Table 1, assuming that the null hypothesis *H*_0_ is true.

**Table 1:** *p*-values calculated by permutation test for every two parts of data.

*p*-value	*A*_1_	*A*_2_	*A*_3_	*A*_4_
*A*_1_	\	0.0684	0.0000	0.0000
*A*_2_	0.0699	\	0.0011	0.0000
*A*_3_	0.0000	0.0015	\	0.0000
*A*_4_	0.0000	0.0000	0.0000	\

Re-sampling is repeated 10,000 times for each *p*-value.

These *p*-values are quite small except that the *p*-value between *A*_1_ and *A*_2_ is slightly higher than 0.05. Small *p*-value means that our observed differences would happen rarely due to randomness. Hence, under the level of 0.05, the difference between *ξ*_1_ and *ξ*_2_ is not significant. While for other pairs of *ξ*, *H*_0_ should be rejected.

**Fig 6:**
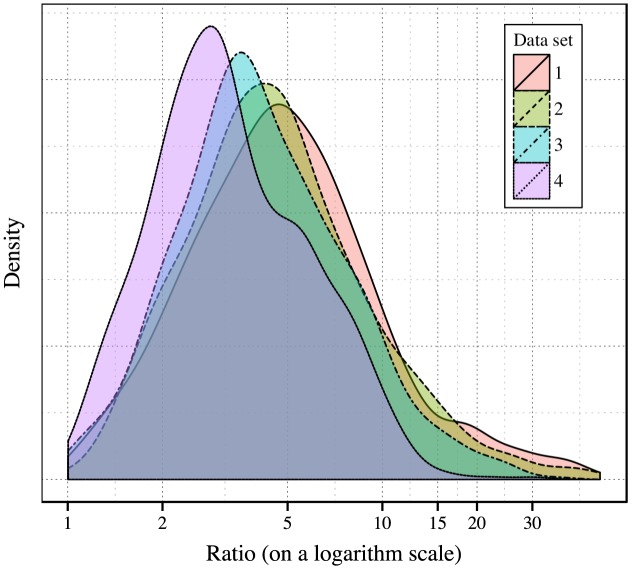
Probability density distributions of *p*_*m*_/*p*_*s*_ for each part of data on a logarithmic scale. For each data set *A*_*i*_, we calculate every *p*_*m*_/*p*_*s*_ in it, and plot the density of the distribution of *p*_*m*_/*p*_*s*_. The density curves are smoothened by one dimensional kernel density estimation.

Secondly, we examine the distribution of our data after logarithm transformation. Fig 6 shows the probability density distributions for each part of data. We use one dimensional kernel density estimation to smoothen the distributions. We then perform Kolmogorov-Smirnov goodness of fit test to check the normality of the transformed values. The K-S statistics are shown in Table 2 under the null hypothesis that each part of data is drawn from normal distribution. When sample size is greater than 35, the critical value for the K-S test statistic has the form:

$$\begin{array}{c} \frac{\sqrt{-0.5\cdot \ln\left(\alpha /2\right)}}{\sqrt{n}},\;n>35 \end{array}$$ (3)

where *α* is the level of significance. For *A*_1_, *A*_2_ and *A*_3_, the null hypothesis cannot be rejected if we choose the significance level as 0.01. While for the fourth part of data *A*_4_, the null hypothesis should be rejected. Considering that all four K-S statistics are relatively small, it is reasonable to conclude that, after logarithm transformation, *p*_*m*_/*p*_*s*_ approximately follows normal distribution.

**Table 2:** Statistics calculated by K-S goodness of fit test for each part of data.

\	*A*_1_	*A*_2_	*A*_3_	*A*_4_
K-S statistic	0.0382	0.0559	0.0483	0.0634
Sample size	795	810	813	807

Based on the normality of the transformed data, once again, we perform *Z*-test to check if there exist significant differences among those four data sets. Since the mean value and the median value of normal distribution are very close, we instead construct the null hypothesis *H*_0_ as: Let *μ*_*i*_, *i* = 1, 2, 3, 4 be the means of the distributions of *A*_*i*_ after logarithm transformation, there are no significant differences between any two means *μ*_*i*_, i.e., *μ*_*i*_ = *μ*_*j*_, *i* ≠ *j*. The *Z*-statistics are shown in Table 3. Since the critical value for *Z*-statistic is 1.96 under 0.05 level of significance, and 2.58 under 0.01 level of significance, the hypothesis *μ*_1_ = *μ*_2_ cannot be rejected. For other pairs of *μ*, *H*_0_ should be rejected.

**Table 3:** Statistics calculated by *Z*-test for every two parts of logarithmically transformed data.

∣*Z*∣	*A*_2_	*A*_3_	*A*_4_
*A*_1_	1.366	4.735	12.238
*A*_2_	\	3.431	11.154
*A*_3_	\	\	7.916

The *Z*-test results are consistent with the results yielded by permutation test. These results suggest that there is no significant difference between the medians of *A*_1_ and *A*_2_. But the differences between other pairs of data sets cannot be simply ignored.

The results of our statistical tests indicate that the robustness of the distribution of *p*_*m*_/*p*_*s*_ is actually a little more complex than what we observed before. Now the question is, how large are the differences between the medians of every two data sets if the differences are statistically significant? In our previous tests, the difference between the medians of *A*_1_ and *A*_2_ is not significant. With regard to the medians of *A*_2_ and *A*_3_, if the null hypothesis is that the relative difference between the medians of *A*_2_ and *A*_3_ is no larger than 5%, the *Z*-statistic is 1.194. It is lower than the critical value 1.645 at the 0.05 significance level, and the null hypothesis cannot be rejected. The difference between the medians of *A*_3_ and *A*_4_ is relatively large. Their values are 3.907 and 3.100, respectively. We believe that this large difference stems from the way we split the data set. Our data is separated by the quartiles of the distribution of saturated forwarding number, which leads to the consequence that almost all the most popular but rare messages are assigned into *A*_4_, since that the popularity distribution of messages is highly skewed. Hence, it is understandable that there exist distinguishable differences between *A*_4_ and any other part of data.

The above results show that for most message samples, the distribution of *p*_*m*_/*p*_*s*_ is robust to the change of message popularity. There exist differences only between the messages with high popularity and those with moderate popularity, and the relative difference is around 20% in our data. In general, the differences among those medians in Fig 5 are not remarkable, and hence we cannot draw solid conclusions on the tendency of the variation of these medians. This general conclusion provides another evidence that the median of the distribution of *p*_*m*_/*p*_*s*_ is relatively fixed. It is robust not only against the differences in network structure, but also against the change of message popularity. Since very popular messages are rare, we are allowed to utilize the relatively fixed distribution to predict the popularity of messages.

### Popularity Prediction

Based on the above results, we propose a parsimonious prediction method to estimate the saturated numbers of forwarding activities. Our prediction is performed on direct follower networks.

**Fig 7:**
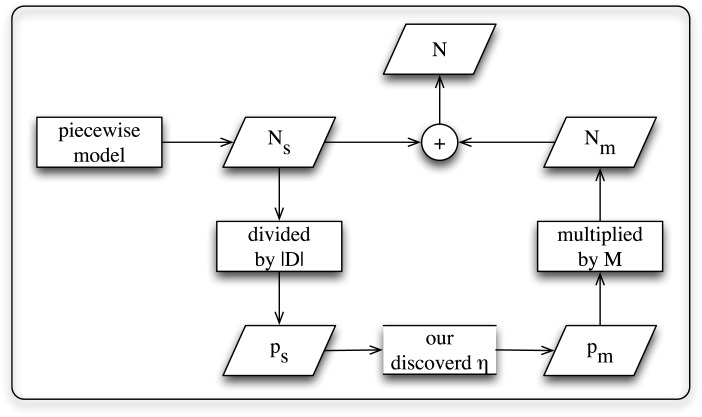
Schematic diagram of our popularity prediction method.

At first, we introduce the basic idea of our prediction method. The mathematical symbols used in our method are listed in Table 4. And the schematic diagram of our method is illustrated in Fig 7. We aim to estimate *N* at a relatively late stage of spreading. This task could be decomposed into the estimation of *N*_*s*_ and *N*_*m*_. *N*_*s*_, the saturated forwarding number contributed by the users who are exposed to a certain message only once, is estimated based on a piecewise model. Then we divide *N*_*s*_ by ∣*D*∣, the number of followers in the entire network *D*, to obtain *p*_*s*_. ∣*D*∣ is a static parameter of the network under research. Since we found a simple relation between *p*_*s*_ and *p*_*m*_, which approximately is *p*_*m*_ = *η* ⋅ *p*_*s*_, where *η* is a constant. We calculate the median of the distribution of *p*_*m*_/*p*_*s*_ in each direct follower network as the value of *η*. This parameter is relatively fixed, but it might be different for different networks. Then, in order to approximately estimate *N*_*m*_, we multiply *p*_*m*_ by the total number of users who are repeatedly exposed to a message at the moment we perform prediction. All the parameters used in this method could be estimated or obtained at the moment we perform prediction.

**Table 4:** Mathematical symbols used in prediction.

Symbol	Description
∣*D*∣	number of all followers in direct follower network *D*
*N*(*t*)	cumulative number a message has been forwarded with respect to time *t* after the original posting
*N*_*s*_(*t*)	cumulative forwarding number contributed by the users who are exposed to a certain message only once
*N*_*m*_(*t*)	cumulative forwarding number contributed by the users who are repeatedly exposed to a certain message
*t*_0_	time at which we perform prediction
*t*_*e*_	time at which there are no more remarkable forwarding activities of a certain message

Specifically, for the users who are exposed to a message only once before forwarding it, there is no reason to expect that these users could obtain extra information other than that from the root user. We separate these users from the others, *N*_*s*_(*t*) denotes their cumulative amount of forwarding activities. By prudently examining the curves of *N*_*s*_(*t*), we discover that these curves satisfy a power function at the early stage of spreading, which is consistent with, though on a small time scale, the findings in [36] and [37]. As dynamical processes approach to saturation, we simply use a linear model to approximate the growth. Hence, the following piecewise model allows us to estimate *N*_*s*_ at a relatively early stage.

$$\begin{array}{c} N_{s}\left(t\right)∼\{\begin{array}{cc} at^{b}+ct,\;a,b,c\in R & \text{if}\;t\leq t_{0}\\ mt+n,\;m,n\in R & \text{if}\;t>t_{0} \end{array} \end{array}$$ (4)

The above piecewise model provides the value of *N*_*s*_(*t*_*e*_). The probability *p*_*s*_ is calculated by dividing the saturated *N*_*s*_ by ∣*D*∣, the number of nodes in the entire direct follower network. And *p*_*m*_ can be obtained by simply multiplying *p*_*s*_ by *η*, the fixed median of the distribution of *p*_*m*_/*p*_*s*_ in a certain network obtained from our data. At time *t*_0_, we can identify all the users who have already forwarded the message, as well as their exposure numbers *σ*_*i*_(*t*_0_). We approximate *N*_*m*_(*t*_*e*_) by *p*_*m*_ ⋅ *M*(*t*_0_), where *M*(*t*_0_) can be calculated by *σ*_*i*_(*t*_0_) according to Eq (2). Hence, *N*(*t*_*e*_) is the sum of *N*_*s*_(*t*_*e*_) and *N*_*m*_(*t*_*e*_). Our method can be summarized in the following steps:

Step 1: At time *t*_0_, estimate *N*_*s*_(*t*_*e*_) based on a power-linear piecewise model.
Step 2: Calculate *p*_*s*_ by *N*_*s*_(*t*_*e*_)/∣*D*∣.
Step 3: Calculate *p*_*m*_ by *η* ⋅ *p*_*s*_, where *η* is a constant that represents the relatively fixed median of the distribution of *p*_*m*_/*p*_*s*_ in a certain network discovered from our data.
Step 4: At time *t*_0_, calculate the number of users who are repeatedly exposed to a certain message, i.e., *M*(*t*_0_) in Eq (2). We approximate *N*_*m*_(*t*_*e*_) by *p*_*m*_ ⋅ *M*(*t*_0_) since that the rate of growth of the forwarding activities in the early spreading stage is larger than that in the late stage.
Step 5: Calculate *N*(*t*_*e*_) by the summation of *N*_*s*_(*t*_*e*_) and *N*_*m*_(*t*_*e*_).

**Fig 8:**
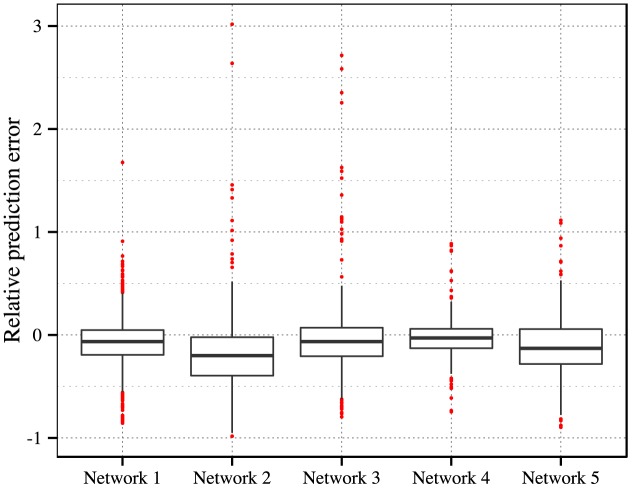
Relative prediction errors for 5 networks with their corresponding root users. These users are the top 5 most active ones in our data.

We use the method mentioned above to predict the saturated numbers messages have been forwarded. The experiments are conducted at the 4th hour of spreading, to estimate the cumulative forwarding number of messages at the 48th hour (our method is not limited to this particular time for prediction). We collect the messages posted by the top 5 most active root users described in Table 5 as our data for prediction. We do not distinguish single-peak patterns from multi-peak ones. Instead, we aim to predict all activities happened on direct follower networks. The boxplots of the relative prediction errors for each network are shown in Fig 8. For networks 1, 3 and 4, the saturated numbers of forwarding activities are well predicted with absolute relative errors less than 25% for more than 77%, 70% and 82% of their message samples, respectively. While for networks 2 and 5, the prediction errors are less than 25% for 50% and 62% of their message samples.

**Table 5:** Details about the direct follower networks in use.

#	Description	# of nodes	# of samples
1	A news magazine	2,371,372	1,265
2	An account for jokes	4,781,102	432
3	An account for witticisms	477,559	361
4	A writer	999,475	313
5	An actress	5,636,055	252
6	A scholar/critic	412,448	212
7	An account for astrology	2,065,209	198
8	An entrepreneur	2,306,919	170
9	A sportscaster	1,938,901	157
10	An account for witticisms	2,694,431	146

We show the numbers of networks, the identities of root users, the numbers of nodes in the corresponding direct follower networks and the numbers of message samples we collect for each root user, respectively.

We recognize that our approach has limitations in predicting the forwarding numbers of root user 2. We have mentioned that almost all the messages posted by root user 2 are obscure jokes. Specifically, people may not get some jokes at the first reading. Some users will add comments and then forward messages. These comments may help others to understand the original jokes. Hence, repeated exposures will play a more important role under these circumstances. The relatively fixed *η*, however, reflects only the average responses in a network. In contrast to more various topics posted by other root users, we believe that it is the particular selectivity that makes the prediction performance mediocre.

**Fig 9:**
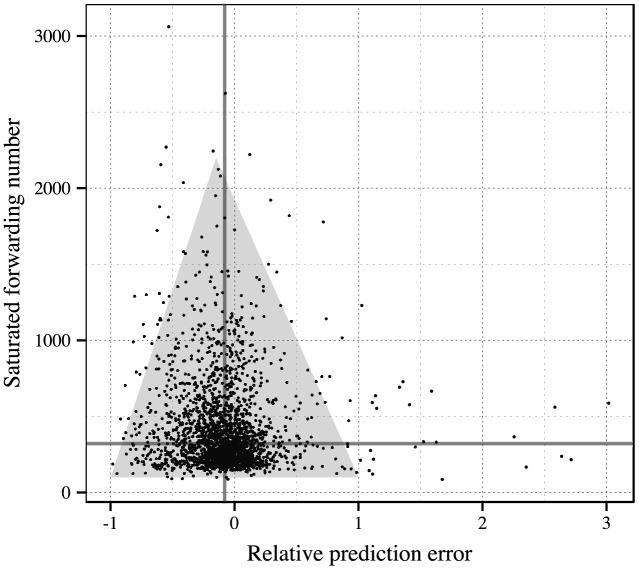
The relation between the prediction error and the saturated forwarding number. The shaded triangular area encloses most points.

In addition, we show the relation between the prediction error and the saturated forwarding number in Fig 9. The shaded triangular area indicates that the samples with large errors are roughly those messages with only small numbers of forwarding activities.

## Discussion

To sum up, we analyze the ratios of the forwarding probabilities under repeated exposures (*p*_*m*_) to the probabilities under only one exposure (*p*_*s*_). Our primary findings are: 1) for those messages with sufficiently various topics, there exists a relatively fixed median of the distributions of these ratios, regardless of the differences in the sizes and structure of networks or the influence of authors; 2) the medians of the distributions of *p*_*m*_/*p*_*s*_ are robust against the change of message popularity. These findings are based on a large-scale and representative data set, as well as reasonable simplifications.

Intuitively, the ratio *p*_*m*_/*p*_*s*_ is a multiplier of the increase in the willingness that people forward messages when repeated stimuli occur, which we believe reflects the increase in the intensity of social reinforcement [21]. Our results provide evidence analyzed from real data. Furthermore, our first finding implies that the increase in the intensity of social reinforcement could be a constant value, and this value is irrelevant to network structure and message popularity. A possible explanation is that this constant reflects the intrinsic psychological gain for average people in adopting or approving new information when multiple stimuli occur.

Our second finding should be interpreted dialectically. Firstly, the variance is observable from a fine-grained perspective between those messages with very high popularity and those with moderate popularity. Those Weibo accounts with specialized topics show higher medians of *p*_*m*_/*p*_*s*_. Obscure jokes (the account of Network 2) may be hard to understand at first. When more and more neighbors add comments and forward these jokes, it is much easier for one to understand and forward them. It makes sense that more popular messages are more probable to capture people’s interest when the very first exposure occurs. This finding provides evidence that the intensity of reinforcement changes with the level of popularity of messages under some circumstances. This finding also provides evidence that topically meaningful communities do exist in social networks [38]. And multiple stimuli may exert more intense impact on some social minority groups. Secondly, the variance could be ignored from a coarse-grained perspective. The distribution of *p*_*m*_/*p*_*s*_ is relatively robust to the change of message popularity. This invariance not only supports our first finding, but also could facilitate related applications. And it is more reasonable to utilize a fixed constant in our prediction method.

Traditional psychological and behavioral researchers put a lot of effort into collecting adequate amount of data and determining causality links. The emergence of online social networks provides large-scale data of individuals’ activities and the connections among them. This is an unprecedented opportunity for traditional psychological problems [18–20]. Our findings may trigger future psychological research on people’s willingness of adopting new things and people’s attitude toward different types of information. Our findings also imply that making better use of the topology of networks for information spreading will be paid back many fold. Hence, influential mediators who are followed by a large amount of users play an important role in informing more people and maintaining messages longer in social medium [39, 40]. These implications may shed light on behavioral and psychological sciences where the mechanism of traversing psychological thresholds for decisions matters, including rumor spreading, innovation diffusion and word-of-mouth in conventional medium. Possible applications of our findings include targeted advertising, recommendation, recruitment campaign and rumor control.

Moreover, several techniques listed as follows, which make our research feasible, can be extended to related studies. 1) We only consider repeated exposures rather than certain numbers of contacts. For instance, we cannot identify whether a user sees a message 5 or 6 times among 10 pushes on his device. Though we can ensure that he repeatedly sees the message with a high probability since the probability that he sees the message exactly once is relatively small. And this compromise does not affect our findings. 2) A message could spread into several layers of a network [1]. If we establish a subnetwork with all possible users who may see the message, the size of the subnetwork may be difficult to deal with. Our definition of *direct follower networks* reduces the scale of our problem significantly and rationally. The scope of the spreading of our original message samples can be decomposed into several these direct follower networks. This definition could be a building block for the research of large-scale spreading, such as cascades. We also observe that only a small amount of influentials are capable of stimulating remarkable peaks since the distribution of users’ followers is highly skewed [41]. 3) Another simplification is the approximation of *p*_*m*_ and *p*_*s*_. In fact, *p*_*m*_ and *p*_*s*_ change after every occurrence of the event of forwarding. The number of users who forward messages is so small (compared to the huge number of followers of an influential root user in a popular microblogging service) that it is not necessary to trace the variances of *p*_*m*_ and *p*_*s*_ dynamically. In addition, forwarding activities will saturate eventually. Hence, if we choose an approximate time window that covers most of the activities, the event-driven property of forwarding can barely effect *S*(*t*) and *M*(*t*). So it is reasonable that the definitions of *p*_*s*_ and *p*_*m*_ are the approximations to the true probabilities of forwarding activities.

The limitations of our work are: 1) although our data sets contain tens of millions of message records, it is still not enough to support the results of calculating the probabilities conditioned on certain numbers of exposures; 2) our prediction method does not use the state of every user and the topology of the networks at the time of prediction. It is designed to show the significance of the fixed median of *p*_*m*_/*p*_*s*_. In the future, it is interesting to explore the probabilities under repeated exposures for different but specialized contents of messages, such as rumors, advertisements and messages attached with pictures and videos. And we may consider the use of sentiment analysis to classify the polarity of message contents [42]. Also, it is promising to establish a more accurate simulation model based on our findings.

## Materials and Methods

### Data Description

The data we collect for the present work consist of two parts, a set of 69 million messages with their corresponding attributes and a set of 3.7 billion directed links among 80 million unique users for the description of network topology. Our data can be found in [43]. Both of them are crawled from Sina Weibo, the most popular Twitter-like microblogging service in China. These data sets are representative compared to other works [1, 2, 27]. We only keep the messages that have been forwarded more than 200 times for our research, because unpopular messages hardly contain any information for analysis. We use the top 10 most active (in the sense of posting original messages) influential users for the purpose of calculating conditional forwarding probabilities. Their corresponding direct follower networks are built and the dynamical processes of spreading of their original messages are traced on these networks. The details of these 10 direct follower networks are demonstrated in Table 5.

### Classification Method

In order to classify single-peak and multi-peak patterns, we adopt a fixed time window with the size of 1 hour, and perform linear regression within each window to fit the curve of cumulative number of forwarding activities of each message with respect to time. We then compare the slopes of the fitted lines between successive windows. Since every sequence of cumulative activities will approach to saturation eventually, the slopes of the fitted lines will approach to zero. For two successive time windows, if the slope of the fitted line in the latter window is larger than that in the former window, the intensity of forwarding activities increases. We define a threshold to identify this growth. If the threshold is too small, the identified multi-peak patterns violate our intuition. If it is too large, the identified single-peak patterns will be ambiguous. We carefully examine the results of several thresholds and decide to choose 30 degree as the value of the threshold.

### Conditional Probabilities Calculation

We restrict the spreading processes of every message within 48 hours, since most spreading processes approach to saturation before this time in our data. For a certain message, all timestamps of the users who forward it are recorded. The number of exposures to this message for a certain user is calculated by examining how many followees of this user have already forwarded this message before his forwarding. Then *σ*_*i*_(*t*), *S*(*t*), *M*(*t*), *p*_*s*_ and *p*_*m*_ are calculated where *t* takes the value of 48(hour). We plot the boxplots for those 3,225 samples whose *p*_*m*_/*p*_*s*_ are greater than 1. We do not distinguish between single-peak patterns and multi-peak ones in calculating conditional forwarding probabilities. It means that these patterns do not affect our findings. In order to discover whether the median of *p*_*m*_/*p*_*s*_ changes with the level of popularity of messages, we mix all the ratios calculated from the samples of all 10 root users altogether, and split them into four intervals by the quartiles *Q*_1_, *Q*_2_ and *Q*_3_ of the distribution of the numbers messages have been forwarded (their values are 281, 429 and 733, respectively). Boxplots are plotted for the ratios in each interval.

### Prediction Method

We collect the original messages from the top 5 most active authors in our data. We keep those forwarding activities contributed by one-degree users (i.e., users in direct follower networks) by using regular expression to parse the forwarding chains marked by particular symbols. We focus on the activities within direct follower networks, and we aim to predict the saturated forwarding numbers. Then we decompose the activities into two parts: 1) those contributed by the users who are exposed to messages only once and 2) those contributed by the users who are repeatedly exposed to messages. For the curves that describe the activities in the first part, we normalize them and fit them by the power function in Eq (4) when time is less than 4 hour as well as by the linear function in Eq (4) when time is larger than 4 hour. The reason we choose 4 hour for prediction is twofold: 1) If the time is too small, it is very hard for prediction. If it is too large, prediction is meaningless since the processes approach to saturation. 2) The length of people’s working time between two breaks in a day is less likely to be smaller than 4 hours. It is significant to choose a prediction time that is around this length of interval, since a larger length will avoid the variances in between two periods. Then we use all the *σ*_*i*_ at the 4th hour and the mean values of the slopes of the linear models to predict the saturated number of forwarding activities at the 48th hour.
